# Hemorrhagic Longitudinally Extensive Transverse Myelitis (LETM) as a First Presentation of Neuromyelitis Optica Spectrum Disorder (NMOSD): A Case Report and Literature Review

**DOI:** 10.1002/ccr3.70081

**Published:** 2025-01-06

**Authors:** Shima Jahani, Fateme Jamshidi, Abdorreza Naser Moghadasi

**Affiliations:** ^1^ Multiple Sclerosis Research Center, Neuroscience Institute Tehran University of Medical Sciences Tehran Iran; ^2^ Razi Hospital Ilam University of Medical Sciences Ilam Iran

**Keywords:** hemorrhagic longitudinally extensive transverse myelitis, longitudinally extensive transverse myelitishemorrhagic LETM, Neuromyelitis Optica Spectrum disorder, NMOSD

## Abstract

Here we present a rare case of hemorrhagic longitudinally extensive transverse myelitis (LETM) as a first presentation of Neuromyelitis Optica Spectrum Disorder. Even though the patient received aggressive treatment, he showed no significant improvement. Our case highlights the importance of prompt intervention in the case of hemorrhagic myelitis and the diagnostic challenges of hemorrhagic LETM.

## Graphical abstract

Brain and spinal cord magnetic resonance imaging of the patient.

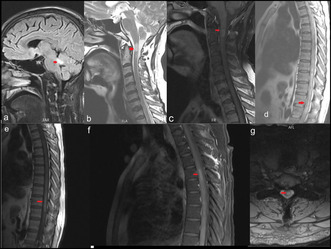

## 1 Introduction

Neuromyelitis optica spectrum disorder (NMOSD) is an autoimmune CNS syndrome with distinct clinical and pathophysiological features from multiple sclerosis. NMOSD, previously known as Devic disease, is commonly associated with serum aquaporin‐4 immunoglobulin G antibodies (AQP4‐IgG) [1, 2].

Recent studies suggest a prevalence of 6.88 NMOSD patients per 100,000 people in United States [3]. Black women are more likely to be affected [4]. NMOSD patients mostly present with lesions of the spinal cord, area postrema, optic nerve, diencephalon, brainstem, and cerebral presentations on MRI findings [5]. The first medication for this condition was approved in 2019, with a commercial name Soliris, a monoclonal antibody that blocks the c5 protein in complement [6]. One year later the second drug for treatment of this condition got FDA approval, inebilizumab, known as Uplizna, which is administered by infusion twice a year [6]. Uplizna is a monoclonal antibody that works by depleting CD‐19 in B cells [6]. The third FDA‐approved medication for this condition is Enspryng, by Genentech, which targets the interleukin 6 (IL‐6) receptor, which is involved in NMOSD neuroinflammation [6].

Acute spinal cord lesions in NMOSD patients are mostly in the form of longitudinally extensive lesions (LETM), defined as lesions longer than three segments. Chronic lesions can be shorter and replaced with atrophy [5]. Even though generally transverse myelitis is mostly presented in a mild form, in very rare cases it can be fulminant, for instance in necrotizing myelitis or hemorrhagic myelitis [7]. Here, for the first time, we describe a case of an NMOSD patient with hemorrhagic LETM. In the absence of clear guidelines, our case would help clinicians to understand how to approach aggressive myelitis in the context of NMOSD disease.

## 2 Case Presentation

### 2.1 Case History/Examination

A 51‐year‐old man presented to the emergency room with a sudden onset paralysis in his lower limbs. He also experienced vertigo, nausea, vomiting, and a severe headache in the frontotemporal region. He had no history of chronic medical conditions, was not taking any medications regularly, and denied any substance abuse, smoking, or alcohol use. He did not experience any recent trauma, fever, or vaccination. Additionally, his family history did not include any autoimmune diseases or neurological problems. During the examination, he was alert and oriented to time, place, and date. Though he looked ill, his vitals were in the normal range. There was nothing significant in his non‐neurologic examination. His force in the right and left upper limbs was four out of five, while his lower limbs had only one out of five. All of his reflexes in both upper and lower limbs were increased. He had a positive babinski reflex in both lower limbs.

### 2.2 Methods (Investigations, Diagnosis, and Treatment)

**FIGURE 1:**
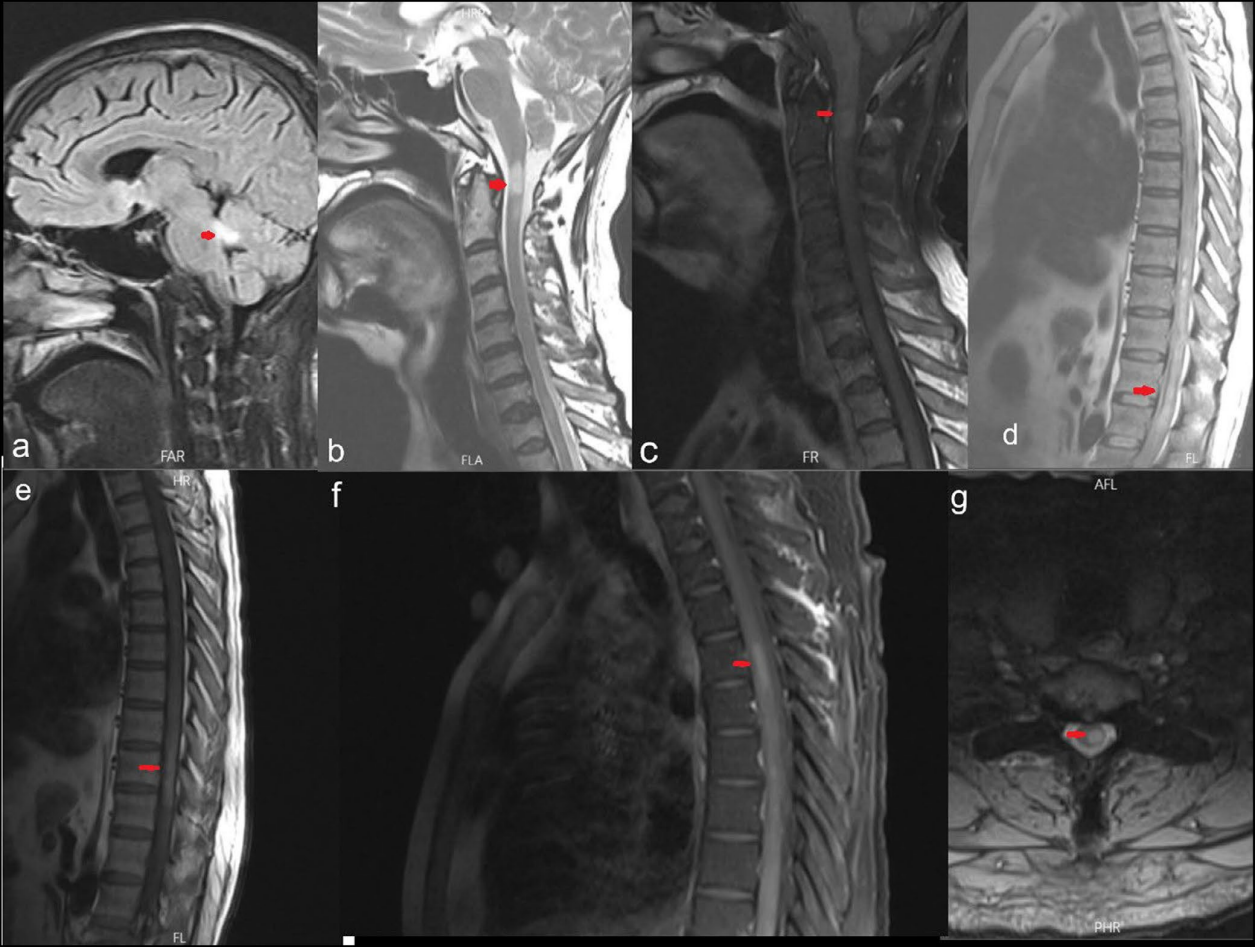
(a) Brain magnetic resonance imaging revealed a third ventricle peri‐ependymal lesion as well as cerebellar peduncle involvement. (b, c) Cervical cord involvement with enhancement. (d) Thoracic MRI revealed hyperintense longitudinal lesion in T2. (e) Imaging showed hyperintensity in T1 which meant hemorrhagic myelitis. (f) Imaging with enhancement. (g) Centrally located hyperintensity has been shown in the axial view of the thoracic cord. Arrows represent the lesions.

After initial brain and spinal Magnetic resonance imaging (MRI), he was diagnosed with myelitis and treated with 4 g methylprednisolone and five sessions of plasmapheresis, in which he showed partial improvement. Shortly after he was infected with COVID‐19, which resulted in Aggravation of symptoms. This time he was treated with 2 g/kg Intravenous immunoglobulin (IVIG). Since he did not show significant recovery, he was referred to our hospital for further treatment. After reviewing his previous brain and spinal MRI, hemorrhagic LETM diagnosis was suggested (Figure 1). Cerebrospinal fluid (CSF) analysis including herpes simplex virus‐1 (HSV‐1), herpes simplex virus‐2 (HSV‐2), Varicella zoster virus (VZV), and Epstein–Barr virus (EBV) was normal. Routine laboratory investigations such as complete blood counts and cerebrospinal fluid evaluations were normal. Venereal disease research laboratory test, vitamin B12 serum level, Human immunodeficiency viruses (HIV), anti‐nuclear antibody (ANA), anti‐coagulopathy, and para neoplastic investigations were normal (Tables 1 and 2). Spinal cord angiography did not show any abnormality. Serum myelin oligodendrocyte glycoprotein (MOG) antibodies were negative, while he had positive AQP4‐IgG. According to his clinical presentation (paraplegia) and imaging (LETM) and AQP4‐IgG seropositivity, the NMOSD diagnosis was confirmed. During his stay in our hospital, he was first treated with IV methylprednisolone 1 g/day for 7 days, which did not result in any clinical improvement. He was then treated with 3 g Intravenous (IV) Cyclophosphamide for 3 days. His corticosteroid therapy was tapered within 10 days.

**TABLE 1:** Laboratory tests of the admission and discharge date.

Lab test (Unit) (Range)	Admission to hospital	Discharge from hospital
White Blood Cell (cells/μL) (4000–10,500)	10,100	9810
Hemoglobin (g/dL) (13.5–18)	13.8	14.5
Platelets (cells/μL) (150000–400,000)	395,000	355,000
Urea (mg/dL) (18–55)	20	25
Creatinine (mg/dL) (0.7–1.4)	1	0.76
Aspartate aminotransferase (U/L) (up to 38)	30	31
Alanine transaminase (U/L) (up to 41)	14	36
C‐reactive protein	positive	negative
sodium (mmol/L) (135–150)	137	138.7
Potassium (mmol/L) (3.5–5)	4.1	3.85

Abbreviations: μL, Microliter; mg/dL, Milligrams per deciliter; mmol/L, Millimoles per liter; U/L, Units per liter.

**TABLE 2:** Cerebrospinal fluid analysis results.

Cerebrospinal fluid analysis (Unit) (Range)	Value
Color	Colorless
Appearance	Clear
Glucose(mg/dL) (45–75)	51
Protein(mg/dL) (15–45)	43.2
White blood cell (cells/μL) (0–5)	0
Red blood cell(cells/μL) (0–1)	0
Xanthochromia	Negative

Abbreviations: μL, Microliter; mg/dL, Milligrams per deciliter.

### 2.3 Results and Conclusion (Outcome)

**FIGURE 2:**
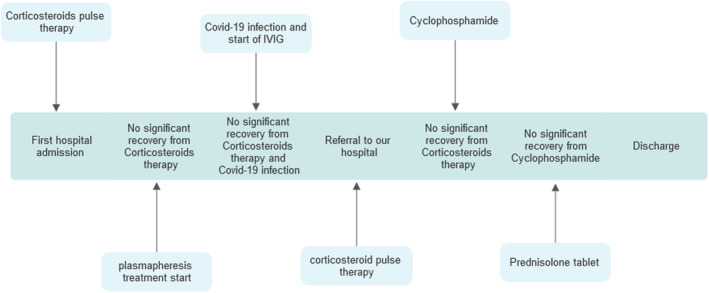
Summarizing patients' hospital visits and treatments.

The patient did not show any improvement during his hospital stay; he was discharged 31 days after his admission (Figure 2).

## 3 Discussion

Spinal cord hemorrhage in context of myelitis is a very rare phenomenon that has been previously reported mostly in post‐infectious settings. Even though there have been very limited documented data on this condition, a case report from back 1915, has focused on histopathological data on hemorrhagic changes of gray matter which mostly happens alongside early necrosis and infiltration of perivascular lymphocytes [8]. The reports focusing on post‐infectious hemorrhagic myelitis events are consisted of cases of different types of Herpes simplex virus, such as HSV‐1, HSV‐2, VZV, and CMV. Also, there are reports of this condition in immunosuppressed patients including cases of HIV and cancer [8, 9, 10]. Noninfectious cases of this condition were very rare and mostly limited to post vaccination scenarios, such as in papilloma and influenza virus vaccination, or autoimmune systemic diseases [9, 11, 12, 13, 14, 15]. Moreover, recently Nerlekar et al. [16], have reported how hemorrhagic myelitis happened in a patient with positive myelin oligodendrocyte glycoprotein antibody. Table 3 summaries the diagnosis, demographic characteristics and outcome of previously published hemorrhagic myelitis cases.

**TABLE 3:** Review of hemorrhagic myelitis cases in medical literature.

Condition	Diagnosis	Age /Sex	Number of cases	Outcome
Post‐infectious	Covid 19 infection	15/ Male	1	Progressive recovery [17]
	Covid 19 infection		3	
		43/Female		No significant recovery [8]
		44/Female		No significant recovery [8]
		26/Female		No significant recovery [8]
Post‐vaccination	Influenza vaccination	33/Female	1	Minimal recovery [11]
	Papilloma virus vaccination	26/Female	1	Partial recovery [12]
Autoimmune diseases	systemic lupus erythematosus	27/Female	1	Partial recovery [9]
	systemic lupus erythematosus	39/Female	1	Progressive neurological recovery [18]
	systemic lupus erythematosus	31/Female	1 (6 cases in total)	Overall recovery [19]
Immunosuppressed patients	Human immunodeficiency virus, VZV infection	41/Female	1	Patient expired [20]
	Human immunodeficiency virus	28/Male	1	No significant recovery [21]
Dimidiating disease	Myelin oligodendrocyte glycoprotein antibody‐associated disease	15/Female	1	Response to treatment and recovery [16]

The presentation of hemorrhagic myelitis on MRI differs in early stages as it is presented as an as isointense lesion on T1‐weighted sequences, later developing to T1 hyperintensity [22]. This presentation progress to T2 hyperintensities in the late subacute phase and lastly T1 and T2 hypo intensity in chronic stages [23]. It is important to differentiate this presentation from vascular or tumoral lesions to choose the appropriate treatment as soon as possible as this phenomenon has a very acute onset and rapid progression and in the majority of cases poor prognosis [9].

AQP4‐IgG is presented in NMOSD patients, this antibody can trigger autoimmune response by complement activation by binding to astrocytes water channels, trigger inflammation and subsequent demyelination [8]. In our case, we hypotheses that the severe inflammation cascade could disrupt vascular integrity and cause bleeding which can cause hemorrhagic LETM.

Hemorrhagic myelitis mostly causes critical fulminant conditions for individuals which differs it from other kinds of mild myelitis such as inflammatory or Demyelinating ones. There are several differential diagnoses involving the condition including: trauma, primary spinal cord tumors, spinal cord involvement by acute hemorrhagic leukoencephalopathy (AHLE), anticoagulopathy, and arteriovenous malformations. This condition can cause severe neurological deficits, mostly accompanied by Plegia. Minimal response to immunosuppressive therapies is one of the most important challenges clinicians are struggling with which unfortunately has caused significant long‐term neurological disability for patients [22].

In our patient, even though aggressive treatment strategies including high‐dose corticosteroids, plasmapheresis, intravenous immunoglobulin therapy, and immunosuppressive agents like Cyclophosphamide were employed, patients' nerve damage became irreversible. This is similar to the results of other cases in literature with different disease backgrounds as most patients did not have significant recovery. Nerlekar et al. [16] report how a 15‐year‐old female, with hemorrhagic LETM and positive MOG antibody with a prompt and aggressive treatment strategy such as IVMP and plasmapheresis, had gradually regained her walking abilities. Unfortunately in our case, aggressive treatment was administered only after hemorrhagic LETM diagnosis which was more than 2 weeks after admission. Another point to discuss is how COVID‐19 could have had effects on aggravating the condition of our patient and his unresponsiveness to treatment.

Our report highlights how prompt interventions could be crucial in the management of patients with demyelinating disorder presenting with hemorrhagic LETM and how clinicians should consider workup for demyelinating diseases such as NMOSD as well as other autoimmune conditions in the management of these patients to prevent irreversible neurological sequelae in affected patients.

## Author Contributions

**Shima Jahani:** conceptualization, data curation, investigation, methodology, visualization, writing – original draft. **Fateme Jamshidi:** data curation, investigation, methodology, resources, supervision, validation. **Abdorreza Naser Moghadasi:** data curation, formal analysis, investigation, methodology, supervision, validation, writing – review and editing.

## Ethics Statement

The authors have nothing to report.

## Consent

The study was conducted in accordance with the Helsinki Declaration, and informed consent was obtained from patients to discuss or publish the details of their disease, their images, and their course of treatment. Informed consent: Written informed consent was obtained from the patient for publication of this case report.

## Conflicts of Interest

The authors declare no conflicts of interest.

## Data Availability

The data used to support the findings of this study are included in the article.
